# Predictive validity of the CriSTAL tool for short-term mortality in older people presenting at Emergency Departments: a prospective study

**DOI:** 10.1007/s41999-018-0123-6

**Published:** 2018-10-31

**Authors:** Magnolia Cardona, Ebony T. Lewis, Mette R. Kristensen, Helene Skjøt-Arkil, Anette Addy Ekmann, Hanne H. Nygaard, Jonas J. Jensen, Rune O. Jensen, Jonas L. Pedersen, Robin M. Turner, Frances Garden, Hatem Alkhouri, Stephen Asha, John Mackenzie, Margaret Perkins, Sam Suri, Anna Holdgate, Luis Winoto, David C. W. Chang, Blanca Gallego-Luxan, Sally McCarthy, John A. Petersen, Birgitte N. Jensen, Christian Backer Mogensen, Ken Hillman, Mikkel Brabrand

**Affiliations:** 1 Centre for Research in Evidence-Based Practice, Faculty of Health Sciences and Medicine, Bond University, Robina, QLD Australia; 2 School of Public Health and Community Medicine, The University of New South Wales, Sydney, NSW Australia; 3 Hospital of South West Jutland, Esbjerg, South Jutland Denmark; 4 Department of Emergency Medicine, Hospital of Southern Jutland, and Institute of Regional Health Research, University of Southern Denmark, Aabenraa, Denmark; 5 Department of Continuous Patient Progress, Bispebjerg og Frederiksberg Hospital, Copenhagen, Denmark; 6 Department of Emergency Medicine, Bispebjerg og Frederiksberg Hospital, Copenhagen, Denmark; 7 Odense University Hospital, Odense, Fyn Denmark; 8 Dean’s Office Dunedin School of Medicine, University of Otago, Dunedin, New Zealand; 9 Ingham Institute for Applied Medical Research, Liverpool, NSW Australia; 10 Agency for Clinical Innovation, Emergency Care Institute, Chatswood, NSW Australia; 11 St George Hospital Emergency Department, Kogarah, NSW Australia; 12 Prince of Wales Hospital Emergency Department, Randwick, NSW Australia; 13 Campbelltown Hospital Emergency Department, Campbelltown, NSW Australia; 14 Campbelltown Hospital Intensive Care Unit, Campbelltown, NSW Australia; 15 Liverpool Hospital Emergency Department, Liverpool, NSW Australia; 16 Sutherland Hospital Emergency Department, Sutherland, NSW Australia; 17 Graduate School of Biomedical Engineering, The University of New South Wales, Kensington, NSW Australia; 18 Centre for Health Informatics, Australian Institute of Health Innovation, Macquarie University, Sydney, NSW Australia; 19 Liverpool Hospital Intensive Care Unit, Liverpool, NSW Australia; 20 South Western Sydney Clinical School, The University of New South Wales, Sydney, NSW Australia

**Keywords:** Risk assessment, Uncertainty, Prognosis, Frail, Aged, Prospective studies

## Abstract

**Abstract:**

To determine the validity of the Australian clinical prediction tool Criteria for Screening and Triaging to Appropriate aLternative care (CRISTAL) based on objective clinical criteria to accurately identify risk of death within 3 months of admission among older patients.

**Methods:**

Prospective study of ≥ 65 year-olds presenting at emergency departments in five Australian (Aus) and four Danish (DK) hospitals. Logistic regression analysis was used to model factors for death prediction; Sensitivity, specificity, area under the ROC curve and calibration with bootstrapping techniques were used to describe predictive accuracy.

**Results:**

2493 patients, with median age 78–80 years (DK–Aus). The deceased had significantly higher mean CriSTAL with Australian mean of 8.1 (95% CI 7.7–8.6 vs. 5.8 95% CI 5.6–5.9) and Danish mean 7.1 (95% CI 6.6–7.5 vs. 5.5 95% CI 5.4–5.6). The model with Fried Frailty score was optimal for the Australian cohort but prediction with the Clinical Frailty Scale (CFS) was also good (AUROC 0.825 and 0.81, respectively). Values for the Danish cohort were AUROC 0.764 with Fried and 0.794 using CFS. The most significant independent predictors of short-term death in both cohorts were advanced malignancy, frailty, male gender and advanced age. CriSTAL’s accuracy was only modest for in-hospital death prediction in either setting.

**Conclusions:**

The modified CriSTAL tool (with CFS instead of Fried’s frailty instrument) has good discriminant power to improve prognostic certainty of short-term mortality for ED physicians in both health systems. This shows promise in enhancing clinician’s confidence in initiating earlier end-of-life discussions.

**Electronic supplementary material:**

The online version of this article (10.1007/s41999-018-0123-6) contains supplementary material, which is available to authorized users.

## Introduction

### Background

Older people with frailty, cognitive impairment and concurrent chronic illness comprise increasing proportions of hospitalizations and emergency department (ED) presentations [1, 2]. Clinicians managing these older people are often faced with the task of recognizing dying and preparing patients and their families for a transition to less active treatment after acceptance of poor prognosis [3]. But uncertainty about chances of survival—almost inevitable in medicine—can delay appropriate management and may lead to non-beneficial interventions [4], social admissions [5], or not medically justified hospitalizations [6].

Predictive tools for older patients [7] may reduce uncertainty but perform inadequately in discriminating risk of death. Some indices have better predictive accuracy but rely on laboratory-based data [8]. Screening for palliative care needs in the ED is reported to be feasible and improve referrals in research settings [9] but it is not a widespread practice in routine care. The Criteria for Screening and Triaging to Appropriate aLternative care (CriSTAL tool) was developed to identify short-term risk of death among a subpopulation of older patients on admission to hospital [10]. The tool was designed based on objective criteria available at the point of care, including the presence of advanced chronic illness, frailty parameters, history of hospital/ICU admission, physiological deterioration criteria, and nursing home residency status (Supplement 1). CriSTAL requires minimal testing (ECG and urinalysis) so clinicians can enhance their confidence in initiating timely sensitive end-of-life discussions on the most appropriate type and place of care for patients nearing the end of life. CriSTAL has been retrospectively evaluated for use in deteriorating inpatients subject to rapid response calls. Frailty was an independent predictor of death within 3 months [11] and a score of 6 has been deemed a flag for high risk of death [12].

### Importance

Prediction of death within 3-months was considered an optimal time as it would give opportunity for more than one discussion as it would not be driving decisions at critical times and would enable patients and/or families to elicit preferences and arrange affairs following discharge. One-year prediction is likely to be modified by factors beyond clinical issues present during hospitalisation. Hence a model that yields accurate prediction of short-term death can inform patients and their caregivers about prognosis, and support management recommendations and shared decision making [13].

### Goals of the investigation

The present study aimed to validate CriSTAL’s accuracy in estimating short-term mortality risk among on a wide range of patients with a variety of chronic illnesses presenting at emergency with exacerbations of chronic disease or injuries. The Australian cohort served as the training cohort and the Danish hospital setting was selected as external validation cohort because they perceived the potential usefulness of CriSTAL in their hospitals after becoming aware of the tool in the development manuscript [10]. This study compared the findings from an Australian patient subpopulation recruited in five teaching hospital EDs in 2015–2016 with a Danish patient population recruited in 2016 from four teaching hospital EDs using the same study protocol [14].

### Primary objectives

1. To determine the ability of the original CriSTAL tool to predict 90-day mortality.
2. To establish the ability of individual and combined parameters in the revised CriSTAL tool to predict death up to 90 days after initial assessment.
3. To determine the minimum number of variables which are sufficient to accurately predict death.

### Secondary objective

4. To determine CriSTAL’s predictive ability for in-hospital death.

## Methods

### Study design and setting

This study is a prospective cohort where older patients presenting at ED were assessed for eligibility and recruited by designated clinical researchers during business hours only in five Australian hospitals (derivation cohort) and four Danish hospitals (validation cohort). Details of design, recruitment, follow-up and analyses are presented in the protocol manuscript, published elsewhere [14].

### Measurements

Documentation of the CriSTAL tool [10] items relating to participants consisted of a designated ED nurse (eight) or medical student (four) searching for the clinical items on the medical record after securing written consent. The tool was translated into Danish and back translated into English language. Questions on any item missing on the medical record were asked of participants in the local language, and data entry also occurred in Danish language. Frailty was integral to the CriSTAL item list, and was measured concurrently with two instruments: the Fried’s frailty phenotype instrument [15] and the Clinical Frailty Scale (CFS) by Rockwood [16], as both have been extensively validated and are known to be associated with future functional decline and death [17]. CFS was determined using clinical judgment based on observation of the patient supplemented with questions on activities of daily living. Fried’s score was determined based on self-report by patient or surrogate given the constrains of movement within the ED. Completion of the CriSTAL checklist took less than 5 min per patient. Baseline and short-term follow-up risk factor data were entered into a web-based interface and transmitted securely to the university server of the faculty where the first author was based. Hospital discharge outcomes were ascertained by the recruiting personnel using a standard template soon after the discharge had been documented by the treating team. Follow-up was conducted by the recruiting nurses via telephone contact with the participant’s household or nominated surrogate’s telephone number (if the participant had died or was unavailable at the time of the call). The intention was to contact them as close as possible to 3 months after initial assessment. However, contact times varied due to patient, family or researchers factors. Details of the design are published elsewhere [14]. The study methods and results are described in accordance with the TRIPOD checklist [18].

### Selection of participants

#### Eligibility

Consecutive older patients (aged 65 years or above) presenting at emergency departments (ED) for any non-elective reason for whom a decision has been made by a doctor to hospitalise or who had spent at least 1 night in the ED. Recruitment conducted over a 6-month study period or until minimum quota sample of 300 was reached in each participating hospital (July 2015 to March 2016 in Australia and January to July 2016 in Denmark). The follow-up period was up to July 2016 in Australia and to November 2016 in Denmark.

#### Exclusions

Patients otherwise eligible but with cognitive impairment, loss of consciousness or inability to speak the local language were excluded if their family/surrogate did not give consent for inclusion or could not act as informant. Patients being discharged on the same day were also considered ineligible.

#### Outcomes

The primary outcome was short-term mortality and the accuracy of this prediction based on the CriSTAL parameters. The secondary outcome was the ability of CriSTAL to predict in-hospital death.

### Analysis

As there is no absolute consensus on the minimal requirement for dataset sample size for modelling, these large contemporary primary data collected on relevant older patients are anticipated to reflect the characteristics of our target population for reproducibility and generalizability of the model [19]. Parameters classified as unknown after medical record check were considered absent. No imputations were used for missing data. Analysis of sensitivity, specificity, and positive/negative predictive value were conducted for individual variables and combinations of variables in backward elimination logistic regression models where the outcome was death at 3 months or in-hospital death. Following our a priori decision, age as continuous variable and sex remained in all models regardless of statistical significance. Results only present the variables retained in the final models. Delays in locating patients for follow-up meant that some outcomes were ascertained beyond 3 months with half ascertained at 4 months in Australia. We hereby refer to the outcome of short-term death as this follow-up period of median 4 months but some participants’ status was ascertained up to 6 months. However, Deaths were not included in the outcome if they occurred after 4 months. Ten thousand random bootstrap resamples were used to internally validate the models and estimate 95% confidence intervals. Areas under the receiver-operating characteristic (AUROC) curve were calculated to determine model discrimination [20]. Calibration was measured by regressing predicted probabilities from the model with the observed values. During the internal validation in Australia using logistic regression directly with CriSTAL score as a summary measure yielded an AUROC of lower accuracy than the model using all the explanatory variables that make up the tool. In the external validation on Danish data, rather than using the summary score we modeled only the association of the CriSTAL components with the outcome. An acceptable model was defined as AUROC ≥ 70% [21]. All tests of significance used *p* < 0.05 (2-sided). All analyses were conducted in SAS version 9.4. Further details of the analysis are presented in the protocol [14].

## Results

### Characteristics of study participants

**Fig. 1:**
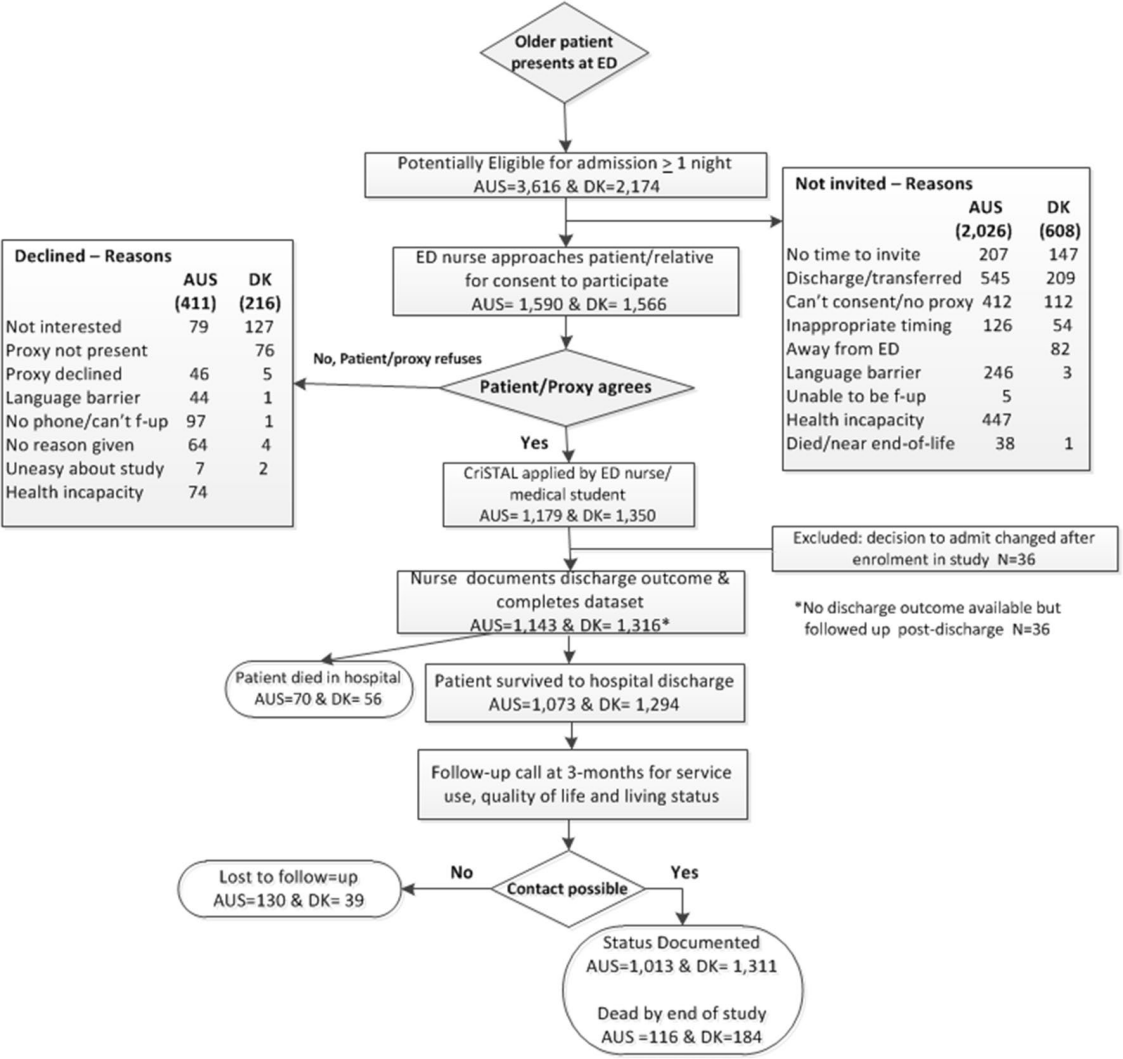
Recruitment and follow-up flow-chart in five Australian and four Danish hospitals

A total of 2493 eligible people (1350 in Denmark and 1143 in Australia) were identified for recruitment only during business hours of 8:00 a.m. to 5:00 p.m. on weekdays due to resource constraints. Some potentially eligible patients left the ED before there was opportunity to enroll them. In Australia 44% potentially eligible could be approached given the large numbers of older patients being admitted including at night, against the limited numbers of recruiters available, while in Denmark the rate was 72%. Details of recruitment, reasons for non-invitation or refusal and extent of follow-up are presented in Fig. 1. Note that in-hospital death or absence or hospital discharge outcome did not preclude the follow-up call to the family for confirmatory information. Thus the final numbers include all who were not lost to follow-up regardless of survival status. Complete follow-up data were available for 88.6% in Australia and 97% in Denmark.

Comparative risk profiles of the derivation and validation cohorts are presented in Table 1. On average the Danish cohort was 2 years younger and was less frail (according to Fried’s scale but not according to CFS), more affected by cancer, CHF and COPD but with similar proportions of people suffering from two or more comorbidities. Yet, the Danish cohort was less likely to have been hospitalized in the previous year, stayed in hospital for longer periods, and had more patients discharged home with community support services. On admission in Australia, females were significantly more frail (Fried score ≥ 3) than males (*χ*^2^ = 9.18, *p* = 0.0024) but not in Denmark (*χ*^2^ = 0.21, *p* 0.64). In both the Australian and Danish cohorts, the prevalence of not for resuscitation orders (4.8% and 4.6%, respectively) and advance health directives was low (0.44% and 1.6%, respectively).

**Table 1:** Comparative risk profiles of the derivation (Australia) and validation (Denmark) cohorts

Risk factor *n* (% of cohort)	Frequency Australia^a^ *N* = 1143	Frequency Denmark *N* = 1350
Median age—years (IQR)	80 (73–86)	78 (76–85)
Female (%)	(52.0)	(53.0)
Median length of stay	3.0 days (95% CI 1.0–7.0)	6.0 days (5.6–6.3)
Nursing home resident	74 (10.3)	139 (10.3)
Advanced malignancy	64 (5.6)	127 (9.4)
Any mental impairment^b^	123 (10.8)	258 (19.1)
Dementia only	70 (6.1)	90 (6.7)
Proteinuria^c^	3 (0.26)	12 (0.8)
Chronic kidney disease	133 (11.6)	70 (5.2)
Fried frailty binary ≥ 1	979 (85.7)	876 (64.9)
Fried frailty score ≥ 3	357 (31.2)	251 (19.6)
CFS frailty ≥ 5	603 (52.8)	702 (52.0)
Congestive heart failure	146 (12.8)	242 (17.9)
Chronic obstructive pulmonary disease	179 (15.6)	327 (24.2)
New or previous myocardial infarction	126 (26.7)	131 (9.7)
New cerebrovascular accident	16 (1.4)	36 (2.7)
Chronic liver disease	19 (1.7)	29 (2.2)
Hypoglycaemia	9 (0.8)	13 (1.0)
Low urinary output	16 (1.4)	5 (0.4)
Abnormal ECG	457 (40.0)	483 (35.8)
Meet ≥ 2 RRS criteria	70 (6.1)	78 (5.8)
Hospital admission in the past year	671 (58.7)	658 (48.7)
ICU admission in the past 12 months	88 (7.7)	98 (7.3)
2 or more chronic conditions	175 (15.31)	218 (16.2)
Community services post-discharge	220 (19.3)	316 (23.4)

Australia: deceased by end of study 116; survived = 1026; Denmark: deceased by end of study = 184; survived = 1166

*RRS* rapid response system deterioration criteria to call an emergency team on admission

^a^Number and % within cohort IQR = interquartile range

^b^Mental Impairment includes one or more of the following: dementia, long term mental illness, disability from stoke, or acute behavioural changes

^c^Information on proteinuria 59% missing in Australia and 41.2% missing in Denmark

There were no differential CriSTAL score or sex distributions between the participants and those lost to follow-up in either cohort, and no age differentials in the Australian cohort. However, the age distribution of the lost to follow-up in Denmark indicated that the youngest group (65–74 years) was more likely to be lost to follow-up (*p* = 0.024).

### Short-term comparisons between survivors and non-survivors

Confirmed participants’ mortality at the end of the follow-up period for all participants was 10.1% (116) in Australia and 13.6% (184) in Denmark. The median follow-up times were 124 days in Australia (IQR 105–170) and 97 in Denmark (IQR 92–149); and most of the deaths occurred within 4 months (84% and 93% at 3 and 4 months, respectively in Australia; and 99% at both 3 and 4 months in Denmark). Those who died in hospital had significantly longer mean length of stay than their surviving counterparts in both health systems.

**Fig. 2:**
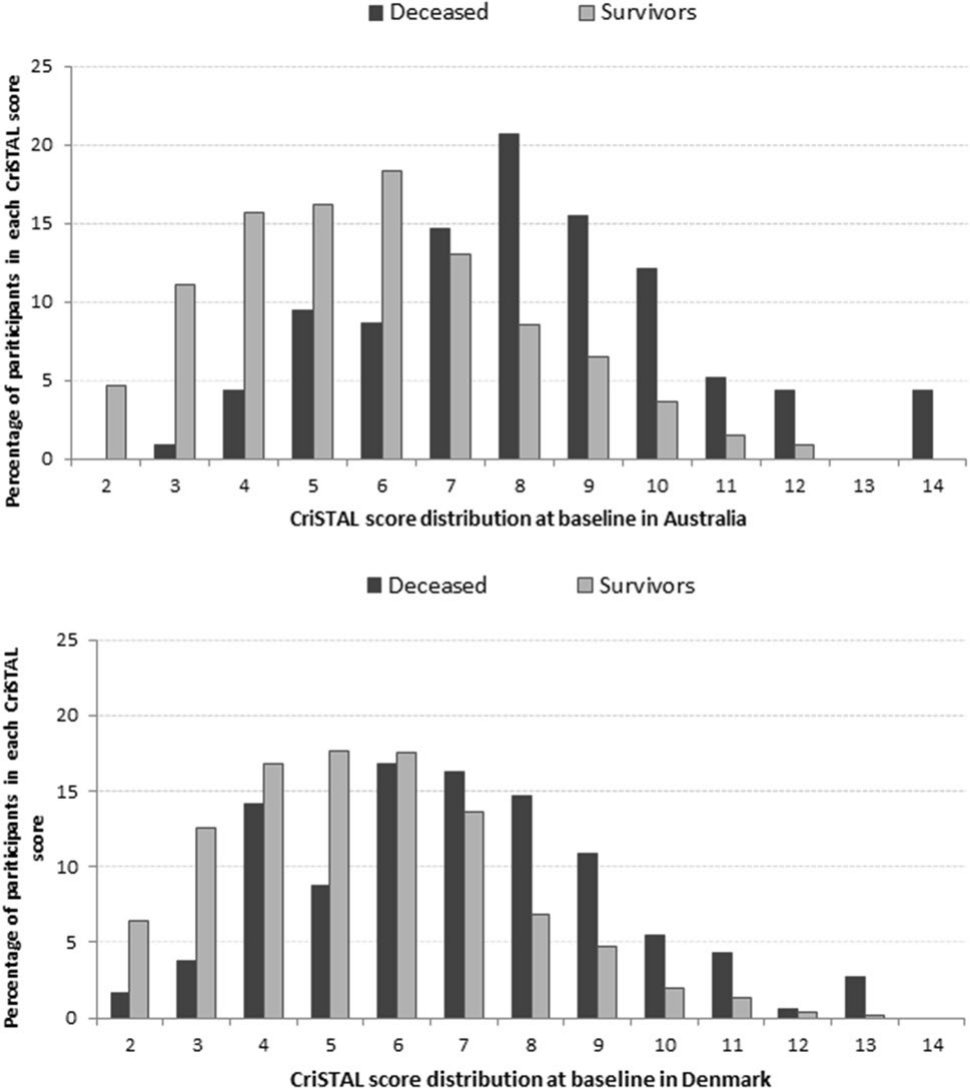
Distribution of CriSTAL scores for all patients by survival status. Inpatients in five Australian hospital (**a**) and four Danish (**b**) hospitals (*N* = 2493)

The deceased were significantly older, mean age 82.5 years (95% CI 80.9–84.1) vs. 79.6 years (95% CI 79.1–80.1) in Australia (*t* test = − 3.68, *p* = 0.0002); corresponding values for Denmark were mean age 81.1 years (95% CI 79.6–82.5) vs. 78.1 years (95% CI 77.6–78.5; *t* test = − 4.30, *p* < 0.0001). In both cohorts the deceased also had significantly higher CriSTAL scores (Fig. 2), with Australian mean 8.1 (95% CI 7.7–8.6 vs. 5.8 95% CI 5.6–5.9) and Danish mean 7.1 (95% CI 6.6–7.5 vs. 5.5 95% CI 5.4–5.6). The deceased were significantly more frail as measured by ≥ 3 Fried parameters (*χ*^2^ = 101.9, *p* < 0.0001), and mean CFS score of 5.6 vs 4.2 (95% CI 5.3–5.9 vs. 95% CI 4.2–4.3, *p* < 0.0001) in Australia; the values for Denmark were ≥ 3 Fried parameters (*χ*^2^ = 157.7, *p* < 0.0001), and mean CFS 6.1 vs. 4.5 (95% CI 5.9–6.4 vs. 95% CI 4.4–4.6, *p* < 0.0001). The range, median and interquartile range of CriSTAL scores in Australia were 2–12, 6 (IQR 4–7) for survivors and 3–14, 8 (IQR 7–10) for the deceased. Corresponding values in Denmark were 2–13, 5 (IQR 4–7) for survivors and 2–13, 7 (IQR 5–9) for the deceased.

### Short-term predictors of death

**Fig. 3:**
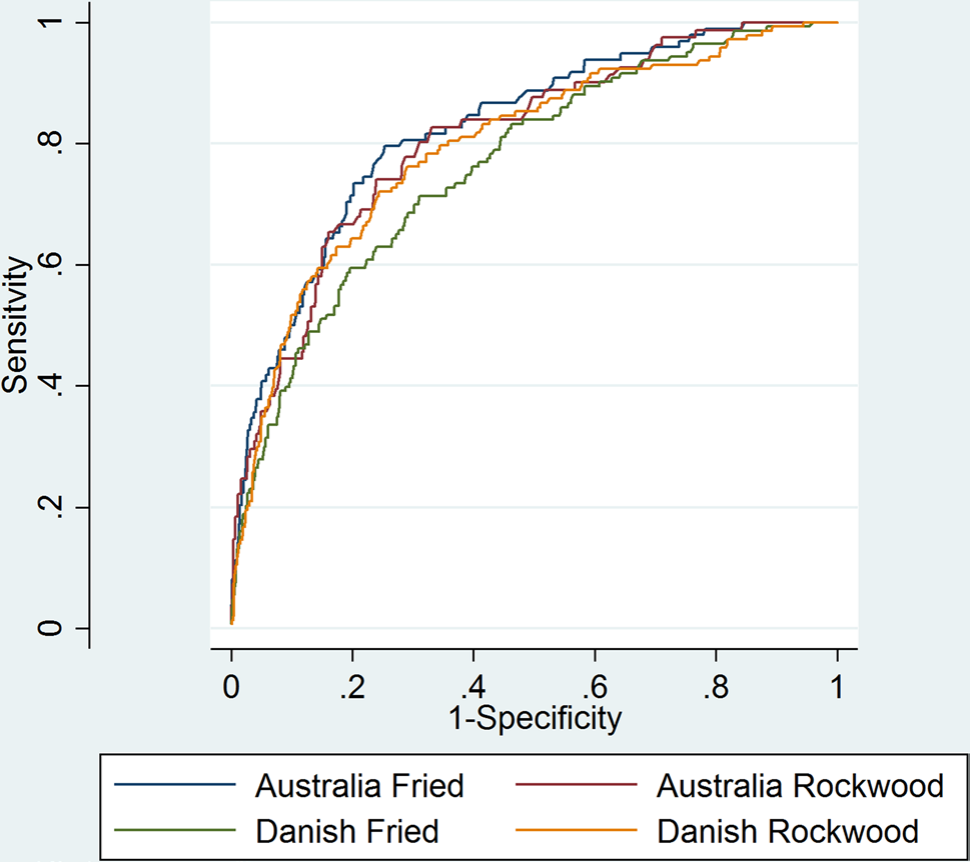
Prediction of short-term mortality: comparison of AUROC for Australian internal validation, and Danish external validation

Applying the Australian CriSTAL algorithm to the Danish data revealed that the Australian model under- predicts the probability of death for the European data (Fig. 3). That is, the model based on patients from five Australian hospitals was (as expected) not optimised for the patient population of four Danish hospitals. Calibration in the large was = 0.76, *p* < 0.001. Given these results, we fitted a model optimised for the Danish data based on their own patient information (Fig. 3 and Table 2 which include all patients with complete follow-up).

**Table 2:** Modelling of short-term mortality using two frailty instruments within the CriSTAL tool for Danish data alone (external validation *N* = 1311) and in the Australian cohort (internal validation *N* = 1013)

A. External validation (DK cohort)	A. DK model with fried frailty scale				B. DK model with CFS frailty scale			
Effect	OR	95% Wald Confidence limits		*p* value	OR	95% Wald Confidence limits		*p* value
Intercept	0.0222	0.0128	0.0336	< 0.0001	0.0042	0.0016	0.0088	< 0.0001
Age	1.06	1.03	1.09	< 0.001	1.05	1.02	1.08	0.0004
Male	2.16	1.50	3.21	< 0.001	1.97	1.34	2.94	0.0006
Advanced malignancy	4.21	2.54	6.91	< 0.001	3.22	1.90	5.44	< 0.001
Oxygen saturation^a^	2.04	1.39	3.01	0.0003	1.56	1.06	2.30	0.0266
Any mental impairment	1.87	1.22	2.89	0.0038				
Frailty as fried	1.52	1.33	1.78	< 0.001				
Frailty as CFS					1.65	1.46	1.90	< 0.001
AUROC	0.764 (0.727–0.810)				0.794 (0.755–0.837)			

B. Internal derivation (Aus cohort)	A. Aus model with fried frailty scale				B. Aus model with CFS frailty scale			
Effect	OR	95% Wald Confidence limits		*p* value	OR	95% Wald Confidence limits		*p* value
Intercept	0.007	0.003	0.014	< 0.001	0.00157	0.00045	0.00404	< 0.0001
Age	1.04	1.01	1.07	0.012	1.04	0.99	1.07	0.0385
Male	2.19	1.39	3.62	0.001	2.45	1.50	4.20	0.0008
Advanced malignancy	5.91	2.89	12.25	< 0.001	4.92	2.19	10.82	< 0.001
Nursing home residence	3.12	1.61	5.91	0.001				
Frailty as Fried	2.15	1.75	2.75	< 0.001				
Frailty as CFS					1.97	1.46	1.81	< 0.001
AUROC	0.825 (0.784–0.869)				0.809 (0.761–0.857)			

Excludes those lost to follow-up by the end of the study

*DK* Denmark, *Aus* Australia

^a^Abnormally low and meeting calling criteria for rapid response call (SaO_2_ ≤ 90%)

After replacing the Fried frailty scale in CriSTAL with the CFS scale, the discriminant ability of the modified CriSTAL model for short-term death in Denmark improved (Table 2). There remained similarity in four of the predictors of short-term outcome between Danish and Australian data: advanced malignancy, frailty, increasing age and male gender In the Australian model advanced malignancy carried a sixfold risk of death, whereas in the Danish model chronic liver disease carried the highest risk of death (fourfold). Frailty was associated with a twofold increased risk of short-term death in Australia and slightly over 50% increased risk in Denmark (Table 2). In both cohorts, CriSTAL as a summary score with Fried as originally designed had lower accuracy in predicting short-term [Danish cohort AUROC 0.687 (95% CI 0.640–0.735) and Australian cohort AUROC 0.768 (0.721–0.814)] death than the accuracy observed when individual CriSTAL parameters were examined.

In both the Australian and Danish models CriSTAL had optimal sensitivity (≥ 90%) at low death probabilities of 3–4% and optimal specificities (> 90%) at higher death probabilities above 20% (see selected cut-off probabilities in Supplement 2).

For the secondary outcome of in-hospital death prediction CriSTAL had only moderate accuracy with AUROC of 0.795 (95% CI 0.737–0.854) for the Australian cohort and 0.682 (95% CI 0.624–0.751) for the Danish cohort (Supplement 3). Sensitivity and PPV were low at probabilities of death of 25% and above, whereas specificity and NPV were generally high at all probabilities of 10% and above (Supplement 4).

## Discussion

This validation study demonstrated that the CriSTAL clinical prediction tool based on existing objective parameters had a good discriminant ability (AUROC 0.81 in Australia and 0.79 in Denmark) for identifying older patients at risk of death up to 4 months in nine hospitals. CriSTAL adds enhanced knowledge by providing a flag for high-risk and an individual probability of death based on personalised risk factors. After controlling for age and sex, a minimum of two variables—frailty and advanced malignancy—were significantly associated with death in both settings. The distribution of CriSTAL scores on admission was higher for those who died than for survivors, indicating its usefulness in enhancing prognostic confidence. As we had used two frailty scales concurrently on admission, an incidental but important finding was that modifying CriSTAL with the inclusion of the CFS frailty scale yielded a better predictive model for the Danish cohort, whereas the original CriSTAL incorporating Fried’s worked better for the Australian cohort. Frailty was also more strongly associated with short-term mortality in Australia than in Denmark, and since administration of the frailty instruments was equivalent, the different impact may be due to the differences in patient casemix or variability in the type of community support provided in the different health systems. This aspect was beyond the scope of our study.

Early identification of frail older people nearing the end of life is important to give patients and families time to prepare and opportunity to discuss values, preferences and avoid unacceptable treatments [22] including potentially preventable emergency re-attendances. CriSTAL is highly specific and different clinicians may choose different cut-off points to initiate end-of-life discussions depending on the predictive probability of death at which their certainty of prognosis is sufficiently reassuring. Advantages of the CriSTAL tool are its objective base, ready availability of parameters, ease and speed of administration (less than 5 min in our experience), and its applicability for non-disease-specific circumstances; it can be applied to a broad range of older patients with multiple chronic conditions admitted to medical or surgical wards. At the same time we acknowledge that it is yet to be determined whether objective prediction tools, even if brief, can be embedded in routine clinical practice in a busy emergency department environment.

In the Australian validation, CriSTAL used as a continuous score was less accurate for predicting death than the model using independent CriSTAL tool components. A model separating the individual components is more useful for clinicians to initiate end-of-life discussions with selected patients about their personalized risk. Worldwide, we would expect that this would be a common minimum set of predictors across different settings. Four of the original 29 variables were predictive of death both in Australian and Danish patients: male gender, increasing age, advanced malignancy and frailty. Other variables may be more or less predictive depending on the impact of health system resources, clinical practices, and patient casemix. Even missing parameters or sampling error can determine which contributing variables will end up in the final optimal model.

Very few clinical prediction rules are evaluated and made readily available at the point of care [23]. While the tool was implemented as a research project, this is the largest prospective study of effectiveness of the CriSTAL tool in predicting individual probabilities of short-term death in a real-life setting. Attempts to identify older patients at risk of death in emergency departments [9] to improve prognostic accuracy are reported in the literature for 30-day mortality [24] or 1-year prediction [25]. For short-term mortality (3–4 months), our model results were better than those of risk stratification tools in France [26], Holland [8], and Japan [27]. Others have also found old age, multimorbidity, increasing age, frailty or cancer as common predictors of 3-month mortality [26, 28–30], but no validation has been reported for some (Table 3). Slightly higher performance on 3-month mortality prognosis was found in USA [31] with a prediction tool that involved blood tests.

**Table 3:** Comparison of CriSTAL performance and predictors with other 3-month prediction tools

Country, date, reference, prediction timeframe	Patient type and predictors	AUROC	95% CI
Australia 2016^a^	65+ years-multimorbidity, ED Male, old age, cancer, frailty	0.81	0.76–0.86
Denmark 2016^a^	65+ years-multimorbidity, ED Male, old age, cancer, frailty, oxygen saturation	0.79	0.76–0.84
France 2015 [26]	Older patients with CKD Male, old age, poor mobility, cancer	0.76	0.75–0.77
Holland 2012 [8]	70+ years, ED patients Disease severity, functional/cognitive impairment	0.74	0.67–0.80
Japan 2015 [27]	Patients on terminal chemotherapy 40 + lab tests: Alb, LDH, neurtrophil count	0.77	N/R
USA 2012 [31]	60+ year with end-stage liver disease Modified frailty index, serum sodium	0.82	N/R
Holland 2016 [28]	Older patients discharged from ED Low oxygen saturation, old age	N/R	N/R
Brazil 2008 [29]	Older patients post-hip fracture Old age, Charlson multimorbidity	N/R	N/R
Italy 2013 [30]	Cognitively impaired older persons Cancer and hospital adverse event	N/R	N/R
USA 2016 [32]	55+ year adults in ED Palliative performance score	N/R	N/R

^a^This study *ED* emergency department patients, *CKD* chronic kidney disease, *N/R* not reported

While four core predictors were common across sub-populations in Denmark and Australia, differences in other predictors due to variations in patient casemix [33], health system policies, and clinical practices [34] across countries limit the transportability [7] of CriSTAL beyond the patient-related factors. The Danish health system provides higher level of community support that facilitates patient management out of hospital whereas discharge to nursing home is avoided. It is possible that this explains why nursing home status was not a significant predictor of death in Denmark. In Australia much of the care at end of life happens in acute care, and nursing home is perceived as an alternative for chronic management of frail older people. Risk-adjustment for post-discharge factors such as non-clinical variables is recommended for fair comparisons of hospital performance across health systems [35], but this was beyond the scope of the present study.

Screening for risk of death and early identification of palliative care needs for advance care planning in emergency departments continues to be an important strategy to deal with the anticipated growth in number of emergency presentations by frail older people [9]. The use of clinical prediction rules to assist shared decision-making can improve process and outcomes when treatment choices are clear, but recommendations are known to be variously applied across clinical contexts [36]. High-risk CriSTAL scores are not intended to dictate management but to flag the need for discussion with patients and families on transition from active to palliative or comfort care when appropriate. The approach should ensure flexibility to accommodate differing priorities and disease trajectories. The next steps for translation of findings from our validation study into practice can include: monitoring of the prevalence of end-of-life discussions after identification of high risk; evaluation of impact of screening on the type and place of care pathways, changes in length of hospital stay post end-of-life discussion, and intensity of health service re-attendances; and doctor/patient/family satisfaction with the use of the risk prediction tool. Investigation of the prevalence of high-risk as measured by CriSTAL among older patients not admitted to hospital are the subject of another study in general practice.

Strengths of this study include heterogeneous groups of patients presenting at hospital emergency departments, almost complete discharge outcomes (99.7%), and high follow-up retention rates and mid-term outcome ascertainment (98%). The data were high quality with minimal missing items on potential predictors other than proteinuria. We used internationally accepted techniques for validation of clinical prediction models [37]. Bootstrapping, involving statistical model building from successive sub-sampling (10,000 times) of the original sample ensured the reliability of results.

### Limitations

The differences in the composition and operation of nursing home, emergency departments and community support between the two countries were not measured or accounted for and this may have played a role in some variations in the findings. The relatively small number of events (deaths) in the cohort in the anticipated timeframe limited our ability to present a wider range of predictive factors and probabilities of death with corresponding sensitivity and specificity. This was likely due to the age eligibility criterion set too low at 65-years of age rather than selection of older participants, and to exclusion of patients with cognitive impairment for practical reasons in real-life survey settings [38]. The potential for selection bias exists as recruitment did not take place at night or on weekends due to resource constraints. It has been previously reported that patients arriving at the ED during those times have a more severe clinical profile and experience higher mortality over the next few days [39]. However, if patients admitted at night or on Sundays were in hospital during weekday recruitment times they were invited and recruited. The impact of the exclusion of these cases in our study may be an underestimation of the accuracy of mortality risk. The proportions of eligible people actually recruited varied between settings given the high demand for services (including nights) in Australia and the limited number of staff recruiting participants. This approach may have missed a different type of patient seeking ED attendance at night or on weekends. The limitation of enrolment during business hours was present for both health systems and it is the nature of research in real-life environments with limited funding to cover all hours. Only small proportions of patients with cognitive impairment including dementia (11% in the Australian and 19% in the Denmark cohort) could be enrolled via surrogates. In retrospect, for larger number of events we could have recruited only sicker individual with higher probabilities of death had we invited 80 + year-olds in the study, but the issue of inability to recruit without a surrogate would have remained. Proteinuria was often absent in Denmark and Australia so it had to be removed from the validation. Chronic disease predictors were based on recorded documentation and variability in completeness of prognostic variables by physicians in routine care may have underestimated actual prevalence of risk factors on admission. Frailty indicators were self-reported rather than measured given the restrictions of mobility and practicalities of demonstrating physical functioning in the hospital ED. The need for subjective self-report rather than measured frailty on admission is a well-recognised issue [40]. It is not uncommon in pragmatic studies in routine care, and its concordance with objectively measured frailty is highly satisfactory [41].

In summary, the CriSTAL screening tool demonstrated good discriminant power in predicting short-term mortality among older patients presenting at hospital emergency services. In the hospital setting, frailty measured with the CFS scale has better discriminant power than Fried’s frailty score. This risk prediction tool is not intended to prescribe treatment decisions. The flags of male gender, old age, advanced malignancy and frailty indicate opportunities for end-of-life discussions, and if the tool can be embedded into routine practice it could enhance clinicians’ confidence in prognosis including recommendations for non-acute supportive terminal care. The threshold at which the discussion is initiated should be one where the risk of harm/benefit ratio of having the end-of-life discussion is optimal. Documenting objective items for the CriSTAL tool could be rapidly achieved using readily available parameters from medical records in routine practice. To optimize predictability we recommend either that risk adjustments for health system influences be incorporated, or predictor variables be examined within health systems rather than extrapolated from foreign algorithms due to these external influences beyond individual patient profiles.

## Electronic supplementary material

Below is the link to the electronic supplementary material.

Supplementary material 1 (DOCX 47 kb)
