# Supplementary material for: Predictive validity of the CriSTAL tool for short-term mortality in older people presenting at Emergency Departments: a prospective study
Source: Eur Geriatr Med. 2018 Oct 31;9(6):891–901. doi: 10.1007/s41999-018-0123-6 (PMC6267649; doi:10.1007/s41999-018-0123-6)
Supplement: Supplementary file 1 — Supplementary material 1 (DOCX 47 kb) [file 41999_2018_123_MOESM1_ESM.docx]

**Supplement 1. Cristal tool and its scoring with Fried, and CFS as concurrently measured**

| **□** | **Age >65** (1 point) | |
| --- | --- | --- |
| **□** | Nursing home resident /in supported accommodation (1 point) | |
| **□** | Meets >=2 RRS selected* calling criteria (1 point if at least 2 abnormal parameters) | |
|  | □ | 1-Decreased LOC: Glasgow Coma Score change >2 or AVPU =P or U |
|  | □ | 2-Systolic blood pressure <90 mmHg |
|  | □ | 3-Respiratory rate <5 or >30 rpm |
|  | □ | 4-Pulse rate <40 or >140bpm |
|  | □ | 5-Need for oxygen therapy or known oxygen saturation <90% |
|  | □ | 6-Hypoglycaemia: BGL 1-4 mmol/L ([23](#_ENREF_23), [24](#_ENREF_24)) |
|  | □ | 7-Repeat or prolonged seizures (>5 minute duration or >=2 per day) |
|  | □ | 8-Low urinary output (<15 ml/hour or <0.5 ml/kg/hour) |
|  | Evidence of frailty: 2 or more of these: (1 point for each abnormal parameter) | |
|  | □ | Unintentional or unexplained weight loss (10 lbs in past year) |
|  | □ | Self-reported exhaustion (felt that everything was an effort or felt could not get going at least 3 days in the past week) |
|  | □ | Weakness (low grip strength for writing or handling small objects, difficulty or inability to lift heavy objects >=4.5Kg) |
|  | □ | Slow walking speed (walks 4.5 metres in >7 seconds) |
|  | □ | Inability for physical activity or new inability to stand |
|  | OTHER RISK FACTORS /PREDICTORS OF HOSPITAL DEATH | |
| **☐**  **☐**  **☐**  **☐**  **☐**  **☐**  **☐** | Personal history of active disease: (1 point for each condition present)  1-Advanced malignancy  2-Chronic kidney disease  3-Chronic heart failure  4-Chronic obstructive pulmonary disease  5-New cerebrovascular disease  6-New myocardial infarction  7-Moderate/severe liver disease | |
| **□** | Evidence of cognitive impairment (mark as many as relevant) (1 point if at least one condition)  ☐ Dementia ☐ Long term mental disorder  ☐ Behavioural Alterations ☐ Mental disability from stroke | |
| **□** | **Previous hospitalisation for at least one night in past year** (1 point if at least 1 hospital admission)  ☐ Yes  ☐ No ☐ Not documented | |
| **□** | Proteinuria on a spot urine sample or >30 mg albumin/g creatinine (1 point) | |
| **□** | Abnormal ECG (Atrial fibrillation, tachycardia, any other abnormal rhythm or >=5 ectopics /min, Changes to Q or ST waves (1 point) | |

Concurrent frailty assessment

| **☐** | Estimated Rockwood score >= 5 (1 point if yes)  (Estimated Clinical Frailty Score 1-9) ________ |
| --- | --- |

**RRS=Rapid Response Systems criteria for deteriorated inpatient needing rescuing*

**Supplement 2. Accuracy of CriSTAL death predictions for short-term follow-up: sample cut-off probabilities for both Australian and Danish final models (incorporating frailty as CFS)**

| **Predictive probability**  **cut-off Australia N=1,013**  **(short-term death)** | **Sensitivity (%)** | **Specificity (%)** | **PPV (%)** | **NPV (%)** |
| --- | --- | --- | --- | --- |
| 0.74^a^ | 4.9 | 99.8 | 66.7 | 91.9 |
| 0.50 | 14.8 | 99.4 | 70.6 | 92.7 |
| 0.25 | 32.1 | 95.8 | 41.3 | 93.9 |
| 0.10 | 66.7 | 80.3 | 23.8 | 96.3 |
| 0.05 | 84.0 | 56.2 | 15.0 | 97.4 |
| **Predictive probability**  **cut-off Denmark N=1,311**  **(short-term death)** | **Sensitivity (%)** | **Specificity (%)** | **PPV (%)** | **NPV (%)** |
| 0.70^a^ | 0.7 | 99.9 | 50.0 | 89.5 |
| 0.50 | 10.5 | 99.1 | 57.7 | 90.3 |
| 0.25 | 39.2 | 93.1 | 40.3 | 92.8 |
| 0.10 | 76.2 | 69.3 | 22.7 | 96.1 |
| 0.05 | 87.4 | 45.5 | 16.0 | 96.8 |

^a^ *Highest probability in this patient sample*

**Supplement 3. Adjusted CriSTAL predictors of in-hospital mortality in the Australian cohort (panel A, N=1,013) and Danish cohort (panel B, N=1,311) based on logistic regression, bootstrap resampling.**

| **Effect** | **A. Model with Australian data**  **OR 95% Wald Confidence Limits** | | | **p value** | **A. Model with Danish data**  **OR 95% Wald Confidence Limits** | | | **p value** |
| --- | --- | --- | --- | --- | --- | --- | --- | --- |
| Intercept | 0.00150 | 0.0004 | 0.004 | <0.0001 | 0.014691 | 0.0053 | 0.033 | <0.0001 |
| Male | 3.09 | 1.74 | 5.99 | 0.0004 | 1.20 | 0.77 | 1.93 | 0.4133 |
| Age | 1.03 | 0.99 | 1.07 | 0.1660 |  |  |  |  |
| Advanced Malignancy | 3.76 | 1.50 | 8.56 | 0.0013 | 2.19 | 1.12 | 3.84 | 0.0100 |
| Frailty as CFS | 1.79 | 1.47 | 2.27 | <0.0001 | 1.22 | 1.07 | 1.42 | 0.0026 |
| Low O2 saturation |  |  |  |  | 2.24 | 1.43 | 3.51 | 0.0006 |
| Chronic liver disease |  |  |  |  | 4.72 | 1.22 | 12.28 | 0.0019 |
| AUROC | 0.799 (0.746 - 0.859) | | |  | 0.682 (0.624-0.751) | |  |  |

# Supplement 4. Accuracy of CriSTAL predictions for in-hospital death: sample cut-off probabilities for both Australian and Danish final models with Frailty as CFS.

| **Predictive probability**  **cut-off Australia**  **(in-hospital death)** | **Sensitivity (%)** | **Specificity (%)** | **PPV (%)** | **NPV (%)** |
| --- | --- | --- | --- | --- |
| 0.66^a^ | 1.8 | 99.8 | 33.3 | 94.3 |
| 0.50 | 8.9 | 99.4 | 50.0 | 94.6 |
| 0.25 | 19.6 | 98.6 | 45.8 | 95.2 |
| 0.10 | 48.2 | 87.7 | 19.6 | 96.5 |
| 0.05 | 73.2 | 71.6 | 13.8 | 97.7 |
| **Predictive probability**  **cut-off Denmark**  **(in-hospital death)** | **Sensitivity (%)** | **Specificity (%)** | **PPV (%)** | **NPV (%)** |
| 0.48^a^ | 0.0 | 99.9 | 0.0 | 93.5 |
| 0.25 | 6.8 | 99.4 | 42.9 | 93.9 |
| 0.10 | 47.7 | 82.7 | 16.2 | 95.8 |
| 0.05 | 68.2 | 53.9 | 9.4 | 96.0 |

^a^ *maximum probability of death in the in-hospital sample*
